# Transformation and Degradation of PAH Mixture in Contaminated Sites: Clarifying Their Interactions with Native Soil Organisms

**DOI:** 10.3390/toxics12050361

**Published:** 2024-05-13

**Authors:** Xiaoyu Li, Shengnan Zhang, Ruixue Guo, Xuejing Xiao, Boying Liu, Rehab Khaled Mahmoud, Mostafa R. Abukhadra, Ruijuan Qu, Zunyao Wang

**Affiliations:** 1 State Key Laboratory of Pollution Control and Resources Reuse, School of the Environment, Nanjing University, Nanjing 210023, Chinaquruijuan0404@nju.edu.cn (R.Q.); 2 Faculty of Science, Beni Suef University, Beni Suef 62521, Egypt; rehabkhaled@science.bsu.edu.eg; 3 Materials Technologies and Their Applications Lab, Faculty of Science, Beni Suef University, Beni Suef 62521, Egypt; abukhadra89@science.bsu.edu.eg

**Keywords:** polycyclic aromatic hydrocarbons, microbial community, ryegrass, natural degradation, transformation intermediates

## Abstract

Soil contamination of polycyclic aromatic hydrocarbons (PAHs), especially caused by the mixture of two or more PAHs, raised great environmental concerns. However, research on the migration and transformation processes of PAHs in soils and their interactions with native communities is limited. In this work, soil samples from uncontaminated sites around the industrial parks in Handan, Hengshui, and Shanghai were artificially supplemented with three concentrations of anthracene (Ant), 9-chloroanthracene (9-ClAnt), benzopyrene (BaP), and chrysene (Chr). Ryegrass was planted to investigate the degradation of PAHs and its interaction with native soil organisms in the constructed ryegrass–microbe–soil microcosmic system. The bacterial and fungal communities in soil were affected by PAHs; their species diversity and relative abundance changed after exposure to different concentrations of PAHs, among which *Lysobacter*, *Bacillus*, *Pseudomonas*, and *Massilia* bacteria were correlated to the degradation of PAHs. On the 56th day, the contents of BaP, Chr, and Ant decreased with the degradation process, while the degradation of 9-ClAnt was limited. Nineteen intermediates, including hydroxylation and carboxylated compounds, were identified. The present research would help clarify the potential interactions between PAHs and native organisms in contaminated sites, providing fundamental information for evaluating the transformation risks of PAHs in the natural environment.

## 1. Introduction

Polycyclic aromatic hydrocarbons (PAHs) are persistent and semi-volatile compounds characterized by two or more fused aromatic rings [1]. They are emitted primarily from combustion sources, such as vehicles and coal-burning plants, through the production and use of petroleum-derived substances. PAHs can accumulate in the soil through atmospheric deposition and surface runoff [2]. Due to the low water solubility, PAHs may persist for a long time in sediments and soils, which are important sinks for PAHs in the environment [3,4,5]. For instance, the total concentrations of 21 PAHs in agricultural soils around coking sites were reported to vary from 294 to 1665 μg/g (dry weight) [6]. In recent years, soil contamination in industrial areas has emerged as a serious environmental problem, and the PAHs in contaminated sites might have great potential risks [7].

PAHs possess carcinogenicity, mutagenicity, and other potential toxic effects, and they have been classified as priority pollutants [8,9]. One-, two- and three-ring PAHs are demonstrated to be acutely toxic, while higher molecular weight PAHs are genotoxic [10,11]. Tong et al. showed that the possibility of carcinogenic risk was 45% if PAH exposure exceeded the acceptable threshold of 10^−6^ (TCR) [12]. When exposed to PAHs for a long time, some human organs, including lungs, skin, esophagus, colon, pancreas, bladder, and women’s breasts, are prone to tumor formation [13,14]. In addition, PAHs could cause embryonic toxicity in laboratory animals by binding directly to estrogen and androgen receptors as anti-estrogens and/or anti-androgens [15]. Moreover, some research has revealed the enhanced toxicity of PAH intermediates formed under natural light irradiation and during biodegradation processes involving soil native microorganisms and plants [16,17]. Therefore, given the high environmental risks of PAHs and the great harm to the human body, it is particularly important to clarify the transformation process of PAHs in contaminated soil sites.

PAHs can exert negative effects on soil microbial diversity and may lead to the total loss of certain microbial species [18,19]. Some soil microorganisms could serve as indicators for PAH stress in soil. Moreover, microbes also play important roles in the degradation of organic pollutants, including PAHs [20]. The biodegradation of PAHs in soil is influenced by soil pH, texture, nitrate nitrogen, water content, and aeration conditions [21,22]. Due to its acceptability, sustainability, and environmental aesthetics, phytoremediation by plant cultivation is considered a promising and environmentally friendly technique to address soil pollution of recalcitrant contaminants like PAHs [23,24,25]. Ryegrass was generally superior to other turfgrasses in terms of bioconcentration of PAHs [26] and can be used to enrich PAHs from soil. Thus, the interaction between PAHs and soil organisms in the soil microcosmic system of plants–microbes–soil is relatively complex. However, few studies focused on the migration and transformation of PAH mixture in contaminated sites and the interactions with soil habitats.

In this work, soil samples from contaminated sites and surrounding background points in Handan, Hengshui, and Shanghai city were collected to construct a soil microcosmic system to investigate the interactions of PAHs with soil native microbial communities and plants cultivated. Four types of PAHs (including chlorinated derivatives), namely anthracene (Ant), 9-chloroanthracene (9-ClAnt), chrysene (Chr), and benzo[a]pyrene (BaP), were selected to simulate the mixed contamination of PAHs in soil. Ant, Chr, and BaP were representative PAHs containing three to five rings, and 9-ClAnt was selected as chlorinated PAHs (Cl-PAHs) to simulate the combined contamination in real soil. The growth status of ryegrass and the diversity of microbial communities were characterized. Then, the effects of plants and soil microorganisms on the degradation of mixed PAHs at different concentrations were studied. Finally, potential transformation products of PAHs in soil were identified by mass spectrometry analysis. This study is of great significance in clarifying the potential impact of PAHs on the primary microbial environment in contaminated sites and can provide a reference for assessing the potential transformation risks of PAHs in the ryegrass–microbe–soil system.

## 2. Materials and Methods

### 2.1. Chemicals and Reagents

Detailed information of chemicals and reagents can be seen in Text S1.

### 2.2. Soil Collection and Pretreatment

Soil samples were taken from three industrial parks in Shanghai (SH), Handan (HD), and Hengshui (HS), with the locations shown in Figure S1 and the detailed sampling sites in Figure S2. The study area is divided into different sampling units based on the actual functional areas of the factory. At the same sampling unit, five sub-samples were collected at a depth of 0–20 cm and then mixed well to provide a composite sample. According to the preliminary test results, soil in the three industrial parks was contaminated with multiple PAHs (see Text S2 for details). To facilitate inter-group comparisons and laboratory testing, surface soil samples (0–20 cm) with low background PAH values below the detection limits, which could be ignored, were collected from each of the three polluted sites for experimentation. The initial concentration of the contamination gradient was established according to the Soil Environmental Quality—Risk Control Standard for Soil Contamination of Development Land (GB36600-2018) in China [27]. Specific physicochemical property parameters of the three soils can be found in Table S1. Considering the original pollution of multiple PAHs in the three industrial parks, three concentration gradients (PAHs-1, PAHs-2, PAHs-3) with the total concentrations of the four PAHs being 50, 250, and 1000 mg/kg (dry weight), respectively, were set to simulate low, medium, and high pollution levels. The specific concentration settings of each PAH are listed in Table S2.

### 2.3. Pot Experiments

Details of the pot experiments, such as pot specifications, soil water content, and sampling methods, are recorded in Text S3. Unseeded pots were set as negative controls to evaluate the removal effect of PAHs by sole soil or native microbial consortium. The sterilized soil without the planting of ryegrass was used as blank control groups to evaluate natural degradation, such as leaching and adsorption. The control groups and experimental groups with PAHs ranging from low to high concentrations were named after sampling sites with the suffixes C1–C3, B1–B3, and P1–P3, respectively. For example, SH_C1, HS_B2, and HD_P3 represent unseeded and sterilized pots with 50 mg/kg PAHs in Shanghai soil, unseeded pots with 250 mg/kg PAHs in Hengshui soil, and seeded pots with 1000 mg/kg PAHs in Handan soil, respectively. The group names of different experimental groups and the experimental groups used in different analyses are organized in Table 1 for the readers’ convenience and understanding. All groups were conducted in duplicate.

### 2.4. Analysis Method

After being collected, the samples underwent freeze-drying at −40 °C. Larger soil particles were gently crushed with a wooden mallet to remove clumps attached to the roots of ryegrass. The remaining soil was further ground, and PAHs were extracted and analyzed from the samples within 12 h. To analyze the PAH content, 0.1 g soil was weighed into a centrifuge tube and ultrasonically extracted for 30 min by 10 mL mixture of dichloromethane and n-hexane (V:V = 1:1). At the 56th day, ryegrass shoots were rinsed three times with ultrapure water, freeze-dried, ground into powder, and weighed 0.1 g to analyze the PAH content following the same procedure as soil samples above. A total of 1 mL of supernatant was sampled for HPLC, HPLC-MS, and GC-MS analyses. The initial concentrations of PAHs in soils of different concentration gradients are listed in Table S3. Detailed information on the analysis process can be found in Text S4, Table S4 and Figure S3.

### 2.5. Microbial Community Analysis

Soil samples from unseeded pots were collected at the end of the pot experiments (56th day) for microbial community analysis. The control soil groups without the addition of PAHs, represented by the suffix “BK”, were also analyzed for comparison. The experimental procedure for DNA extraction and PCR amplification are detailed in Text S5. The amplified products were subjected to fluorescence quantification using a BioTek (FLx800) microplate reader (Santa Clara, CA, USA) and then submitted to the Illumina platform for sequencing through Yanqu Information Technology Co., Ltd. (Hangzhou, China).

## 3. Results and Discussion

### 3.1. Effect of PAHs on Ryegrass

#### 3.1.1. Variation in Growth Height of Ryegrass

**Figure 1:**
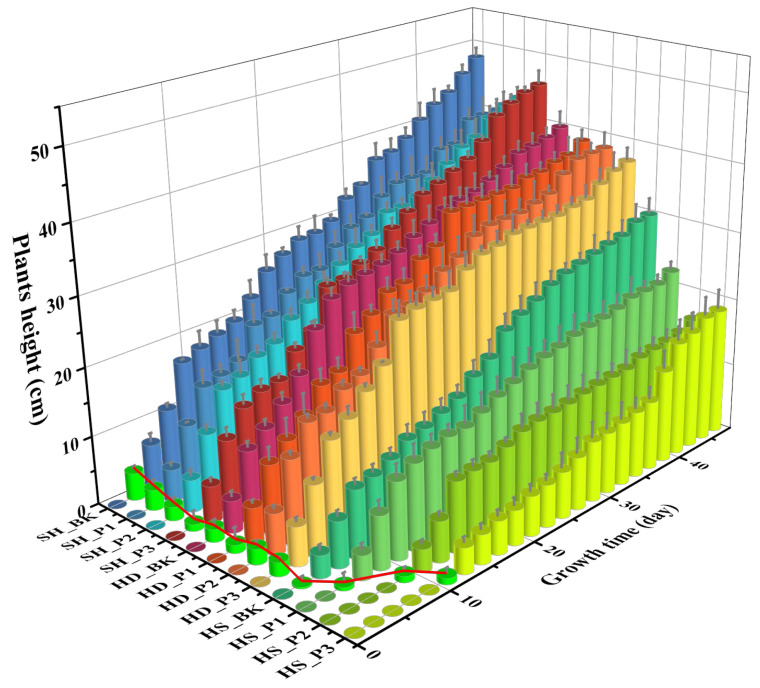
Growth height of ryegrass in soils with three contamination levels of PAH mixture and the germination time (red line). (SH: Shanghai; HD: Handan; HS: Hengshui; BK: Control group without contamination; P1: 50 mg/kg; P2: 250 mg/kg; P3: 1000 mg/kg).

Plant height is the most intuitive physical quantity reflecting the growth status of plants. The height of ryegrass in different soils with three contamination levels of PAH mixture was recorded as a function of growth time (Figure 1). Obvious differences in plant height were observed in different sites, among which ryegrass grew best in SH soil, followed by HD and then HS soil. HS soil took the longest time for seed germination. Ryegrass seed germinated on the third day in SH and HD soils, while the germination occurred on days 3, 5, 9, and 11 in HS soil with total PAH concentrations of 0, 50, 250, and 1000 mg/kg, respectively (the red connecting line). The cultivation of ryegrass lasted for 56 days; the plant height increased slowly, and the growth was relatively stable from the 25th day. Among the three experimental groups, plants in Shanghai soil were stout and tall, with the height ranging from 40 to 46 cm. In Handan soil, plants in the HD-P3 group grew the slowest. However, the final height of plants in the three groups showed no obvious difference, ranging from 36 to 38cm. The worst growth status of ryegrass was observed in HS soil, where the plant leaves were thinner, and the height varied between 16–30 cm. There are multiple heavy metals (e.g., Hg, 176.96 mg/kg) and other organic pollutants (e.g., CCl_4_, 18.99 mg/kg) in the soil, which exceeds the risk screening values for soil contamination of development land prescribed by GB 36600-2018 (China) [27], exerting an adverse effect on plant growth.

#### 3.1.2. Migration of PAHs into Ryegrass

Contents of PAHs in the ryegrass on the 56th day were measured. Due to the poor growth of ryegrass, it was difficult to determine the concentration of PAHs in plants in HS groups. Therefore, only the migration of PAHs into plants in HD and SH soils was studied. As the initial soil PAH content increased, the concentration of three unchlorinated PAHs in plants increased to varying degrees (Table S5). As the initial pollution concentration increased, the concentrations of Ant and BaP in HD ryegrass shoots increased from 0.36 and 0.24 mg/kg to 1.14 and 1.17 mg/kg (dry weight), respectively, representing increases of 0.78 mg/kg and 0.93 mg/kg. For SH groups, the Ant and BaP increased by 0.10 and 0.41 mg/kg from the SH_P1 group to the SH_P3 group. According to a previous study, the initial soil PAH concentration directly affects the accumulation of PAHs in ryegrass [28]. Due to the higher initial concentration in the soil, the concentration of Chr accumulated in ryegrass is higher than that of the other three PAHs. The contents of Chr in PAHs-3 groups reached 2.18 and 6.34 mg/kg in HD and SH soils, respectively. However, the content of 9-ClAnt in plant shoots increased first and then decreased in SH soil, showing a decreasing trend in HD soil. This abnormal phenomenon might be related to the properties of Cl-PAHs. The quantitative structure–activity relationship and level III fugacity model revealed that Cl-PAHs tend to be more enriched in soil and sediment instead of air and water bodies [29]. In addition, the Cl-PAHs in soil and sediment are hard to migrate to soil organisms and to be utilized by plants. The concentration of Cl-PAHs, especially those with larger molecule weight, is much higher in the sediment than in mussels of benthic organisms [30].

### 3.2. Effects of PAHs on Soil Microbial Community Structure

Microorganisms, as an important component of soil ecosystems, are affected in species composition and community structure by the stress of PAHs [31]. As previous studies have demonstrated, contaminants in soil systems could affect the composition of microbial communities [32]. In this section, the changes in soil bacterial and fungal community structure under different PAH stress conditions were studied. The effectiveness of the sequencing results is listed in Table S6. The proportion of valid sequences obtained after quality control, denoising, assembly, and chimera removal exceeds 90% for all experimental groups, except for HD_P1, which was 88.8%, indicating the validity of the sequencing results.

#### 3.2.1. The Alpha Diversity Index Analysis

Alpha diversity, indicated by the Chao1, Shannon, Simpson, Observed Species, and Goods’ Coverage indexes, was used to reveal the species richness and diversity of microbial communities in three different soils (Table S7). Chao1 and Observed Species can characterize the richness while excluding the impact of each species’ proportion (Species Evenness) [33]. As the total PAH concentration increased to 50, 250, and 1000 mg/kg, the Chao1 index of bacteria in the SH soil samples changed from 418.8 (SH_BK) to 970.2, 554.0, and 530.2, respectively. The number of observed species showed a similar trend that increased from 418 (SH_BK) to 969, 553, and 528, respectively. Thus, a low level of PAH pollution greatly increased the microbial diversity of SH soil samples. The PAHs could have a dual effect on microbial communities. When the concentration of PAHs is low, it might change the dominant species in the soil, leading to an increase in the abundance of other microorganisms, especially PAH-degrading bacteria [34]. However, high concentrations of PAHs may inhibit the growth of microbial communities and reduce species abundance. Moreover, the relative abundance of species is also related to the physiochemical properties of the soil, and thus, different types of soils showed different PAH stress tolerance [35]. The Chao1 and Observed Species of bacteria in HD soil samples were higher than those in Shanghai and decreased from 6035.1 (HD_BK) to 1552.0, 1347.9, 1320.6, and from 5404 (HD_BK) to 1545, 1302 and 1316, respectively, with increasing soil PAH concentration.

In the case of fungi, the species richness had negligible variation in SH soil, of which the Chao1 increased from 95.0 (SH_BK) to 105.3, 108.9, and 101.1. However, the two indexes both showed an obvious decrease in HD soil samples, which demonstrated that HD soil was more susceptible to PAHs, resulting in a decrease in species richness. The decreased biodiversity of HD soil might exert an adverse effect on the degradation of PAHs. After exposure to 1000 mg/kg PAHs, the species richness of fungi decreased in HS soil.

The diversity of the microbial community was represented by Shannon [36] and Simpson [37] indices, which both have been traditionally used to measure diversity. The Shannon index is a measure of species richness, while the Simpson index focuses more on species evenness [38]. According to Table S7, both the Shannon and Simpson indexes indicated that the microbial diversity increased in the SH soil sample contaminated by PAHs, while opposite trends were observed in the other two kinds of soils. Good’s coverage [39] was used to evaluate the coverage quality of sequencing results. This index varied from 0.98 to 1.00, suggesting that the sequencing results are representative of the real microbial communities.

#### 3.2.2. Rank Abundance Curve Analysis

The rank abundance curve (RAC) was used to represent the distribution of OTU abundance, and the flatness of the broken line reflects the evenness of community composition. The flatter the line, the smaller the abundance difference among OTUs in the community, and the higher the evenness of community composition [40]. In SH soil samples, the curve became flatter after PAH contamination, especially for SH_P1 (Figure S4a), indicating the increased evenness. HD soil samples showed different trends, with the HD_BK group showing the flattest curve, and almost the same degree of steepening was observed in the curves of the contaminated samples (Figure S4b). Due to the differences in the community composition of the initial microorganisms (see details in Section 3.2.3), the two types of soils responded differently to the secretions of ryegrass. On the one hand, rhizosphere secretions could promote the activity of soil microorganisms [41]. On the other hand, rhizosphere secretions may cause competition by stimulating microbial activity, thereby affecting species richness and composition uniformity [42].

Thus, PAH contamination disrupted the evenness of community composition in HD soil, which had little relationship with the concentration of PAHs. Due to the low species abundance in HS soil samples, its RAC is not displayed. From the analysis of alpha diversity indices and RAC, the microbial abundance and diversity of bacteria were larger than those of the fungal community, which indicated that the bacterial community was dominant in the three soils.

#### 3.2.3. Comparison of the Species Composition in Soils

The differences in bacterial communities among three different soils (SH, HD, HS) were analyzed via PCoA based on the OTUs of the bacterial communities [43]. In Shanghai soil samples, Axis 1 explains 63.8% of species variation (Figure S5a). The SH_P1 group was notably distant from others on Axis 1, indicating that even low PAH contamination could change Shanghai soil community composition. Combining the analysis from Section 3.2.2, the SH_P1 group exhibits higher evenness in species composition. With increasing PAH stress, there might be dominant species adapted to PAH stress. Similarly, in Handan soil, Axis 1 accounts for 49.2% of the variation, with PAH-exposed samples projecting further than blanks, indicating PAH-induced microbial community differences. In Hengshui soil, Axis 1 and Axis 2 explained 43.6% and 31.4% of the variation, respectively, with a cumulative explanatory power of 75.0%. P1, P2, and P3 are closely projected on both Axis 1 and Axis 2. Therefore, PAH mixture altered the composition of indigenous microbial communities in the soil.

The identification results of species composition analysis are listed in Table S8. HD soil samples showed the largest number of bacteria species, with 18–20 phyla, 40–56 classes, 81–111 orders, and 185–269 genera identified. Shanghai soil samples showed increased species numbers after exposure to PAHs. The lowest species numbers were found in HS soil samples, where the number of genera for bacteria and fungi ranged from 10–59 and 3–10, respectively.

**Figure 2:**
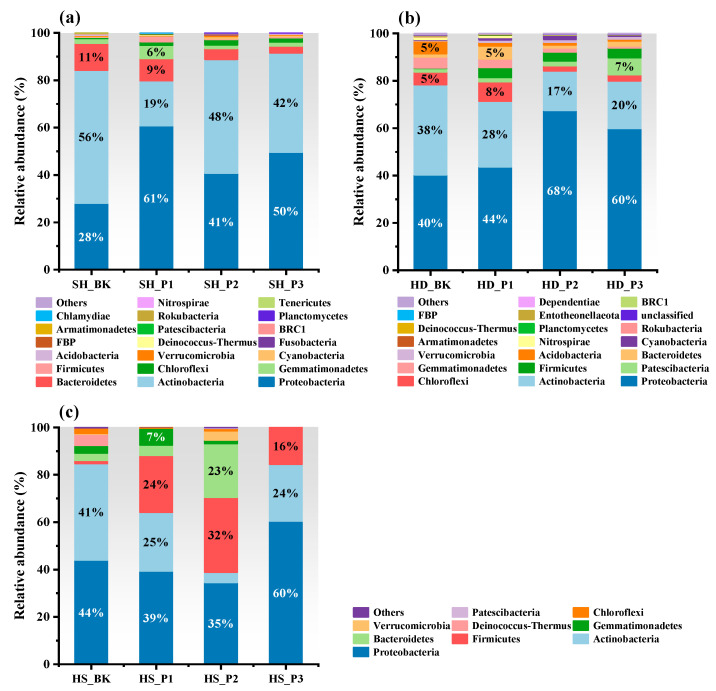
Relative abundance of bacteria at the phylum level in Shanghai (**a**), Handan (**b**), and Hengshui (**c**) soils with different concentrations of PAHs. (SH: Shanghai; HD: Handan; HS: Hengshui; BK: Control group without contamination; P1: 50 mg/kg; P2: 250 mg/kg; P3: 1000 mg/kg).

The composition of bacteria in the phylum level is shown in Figure 2. The dominant bacteria in the three soils were *Actinobacteria* and *Proteobacteria*, the total proportion of which could reach even up to 92%. With the increase in PAH concentration, *Proteobacteria* abundance in Shanghai soil increased by varying degrees from 28% to 61%, 41%, and 50%, while that of *Actinobacteria* decreased from 56% to 19%, 48%, and 42%, respectively. The two microorganisms showed similar variation trends in the soil of Handan, where the appearance of *Firmicutes* was also noticed (4.2%, 3.8%, and 4.1%) after the addition of PAHs. Accompanied by a decreased abundance of *Acidobacteria*, the percentage of *Firmicutes* increased obviously in Hengshui soil. However, only the HS_P3 group showed a higher proportion of *Proteobacteria* than in uncontaminated soil. Although the specific organisms underwent different changes in different soils, the diversity of bacterial composition at the phylum level reduced with increasing PAH concentrations. Only *Firmicutes*, *Actinobacteria*, and *Proteobacteria* were found in the 1000 mg/kg PAH-contaminated Hengshui soil, which might result from the combined effect of PAH stress and soil physicochemical properties. Further analysis was conducted on the composition of bacterial communities at the genus level (Figure S6). The natural PAH-degrading bacteria in soil mainly belonged to *Pseudomonas*, *Bacillus*, *Sphingomonas*, *Lysobacter*, *Rhizobium*, and *Ohtaekwangia* [44,45,46,47,48]. In Shanghai soil (Figure S6a), the abundance of *Pseudomonas* increased in the SH_P1 group (32.4%) and subsequently decreased to 3.6% and 6.4% in the SH-P2 and SH-P3 groups, respectively, with that of SH_BK group being 5.1%. *Sphingomona* showed decreased abundance, which accounted for less than 0.6% of the three PAH exposure groups. For HD soil, the abundance of *Lysobacter* and *Bacillus* increased from 1.9% to 11.1%, 26.9%, and 20.0% and from 0.1% to 1.8%, 2.3%, and 2.4%, respectively with increasing PAH concentrations (Figure S6b). In both Shanghai and Handan soils, the abundance of *Massilia* increased with the increasing gradient of PAH mixture, suggesting its potentiality in PAH degradation, which was consistent with a previous study [49]. In Hengshui soil, both *Bacillus* and *Pseudomonas* increased to become the dominant population after the addition of PAHs, while the proportion of *Nocardioides* was significantly decreased (Figure S6c).

Fungal-mediated bioremediation of PAHs in soil has also been widely reported in recent years. Fungi do not utilize PAHs as their sole source of carbon as bacteria but co-metabolize PAHs and produce a series of oxidation products, including carbon dioxide [50]. Therefore, the abundance changes of fungal communities in different soils before and after PAH pollution were compared (Table S9). The dominant genus in SH_P1 soil was *Ascomycota*, which accounted for up to 53.0%, much higher than that in SH_BK (10.8%). However, as the loading concentration of PAHs increased, its proportion decreased to 26.1% and 16.7% in the SH_P2 and SH_P3 groups, respectively. Other fungal communities have also undergone varying degrees of changes under the influence of PAHs, as detailed in Table S8.

To sum up, PAHs could vary the composition and structure of soil microorganisms. Some bacteria and fungi that have been proven to have the ability to degrade PAHs showed increased relative abundance as PAH concentration increased. However, the abundance of these microorganisms does not always show a positive correlation with PAH concentration. Instead, their abundance increased at low concentrations of PAHs while decreasing as the PAH concentrations were further increased.

#### 3.2.4. Comparative Cluster Analysis

In the sample clustering analysis, UPGMA clustering is performed on samples according to Euclidean Distance of species composition data, and the samples are then arranged based on the clustering results. The abundance information of the top 50 genera in the samples was selected to draw the heat map (Figures S7–S9), from which obvious differences could be found in the distribution of dominant genera. As seen in Figure S7, the dominant genera were distributed in the upper left of the heat map for HD_BK and changed by exposure to different concentrations of PAHs. *Devosia*, *Actinotalea*, *Phenylobacterium*, *Massilia*, *Sphingoaurantiacus*, *ANPR*, and *Shinella*, some of which have been proven to be quite capable of degrading PAHs in the soil or promoting plant growth, showed increased abundance with increasing PAH exposure concentrations [51,52]. By contrast, some of the bacteria, such as *Luteimonas*, *Nitrosospira*, *Longimicrobiaceae*, *Cupriavidus*, and *Anaeromyxobacter*, showed higher abundance in HD_P1 than other groups, indicating that PAH stress at a low concentration was beneficial to the growth of the bacteria to some extent.

The heat map in Shanghai soil of different PAH exposure concentrations also showed differences (Figure S8). *Georgenia*, *Actinotolea*, and *Nitrosospira* were not found in the SH_BK, but their relative abundance increased in the PAH exposure groups. Previous findings have also revealed that the stress of PAHs could lead to an increase in microbial abundance [53], which may be due to the decline of some dominant species [33]. It is noteworthy that in both Shanghai and Handan soils, *Devosia*, *Nitrosospira*, *Actinotalea*, *Luteumonas*, *Paracoccus*, and *JG30-KF-CM45* showed quite a low abundance in the control group but increased to different degrees in the PAH exposure groups. The results of heat map analysis from Hengshui soil samples (Figure S9) showed that there were significant differences in microbial abundance among the four experimental groups, and only a few genera of microbe displayed absolute predominance. The sandy soil in Hengshui was not conducive to the survival of microorganisms; therefore, the abundance of both bacteria and fungi was low.

### 3.3. Effect of PAHs on Ryegrass

**Figure 3:**
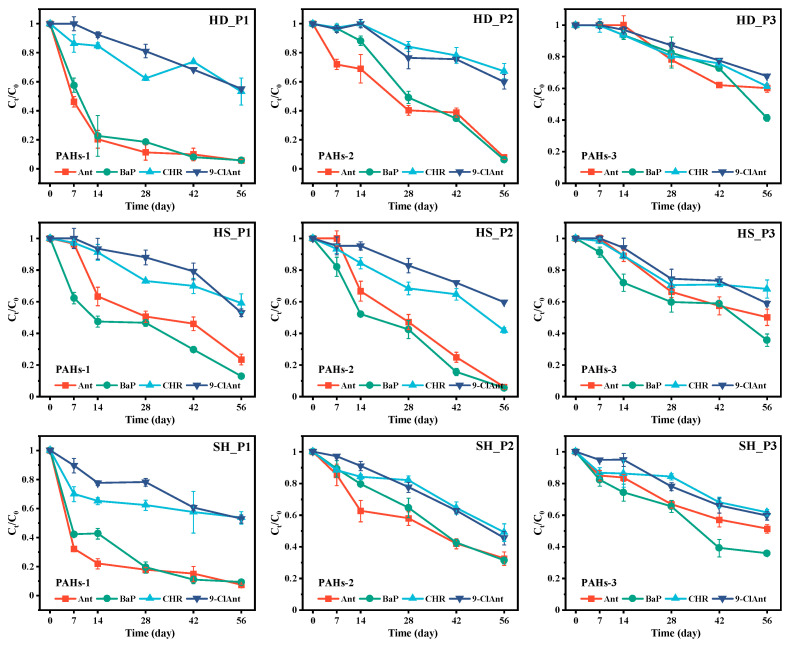
Effects of different PAH concentrations and different types of soils on the degradation of four PAHs in the ryegrass–microbe–soil systems under sunlight irradiation. (C_t_/C_0_ represents the residual concentration variation of PAHs in soil over time; P1: 50 mg/kg; P2: 250 mg/kg; P3: 1000 mg/kg).

The transformation of PAHs in soil is the result of many environmental factors, such as light, microorganisms, and plants. Figure 3 shows the degradation of the four PAHs in the vegetated soils, reflecting the comprehensive effects of plants, microorganisms, and light in the soil system. The residual PAHs all decreased with time in the soils studied. In general, the removal efficiency of the four PAHs was lowered with the increase in the total exposure concentration. The removal percentage of Ant, BaP, Chr, and 9-ClAnt decreased from 94%, 94%, 45%, and 45% in the HD-P1 group to 40%, 59%, 39%, and 32% in the HD-P3 group, respectively. As the total PAH concentration increased from 50 mg/kg to 1000 mg/kg, the removal of Ant decreased by 55.5%, 26.6%, and 41.25% in HD, HS, and SH soils, respectively. The initial concentration of PAHs in soil influenced microbial degradation [54]. The high concentration of PAHs may have an adverse effect on the microbial community structure and plant growth in the soil, which was unfavorable for the elimination of PAHs by soil biota, thus leading to decreased removal efficiency in PAHs-3 groups. Among the four PAHs, Ant and BaP showed better removal effects than the other two PAHs. Specifically, around 85% of Ant and BaP were removed in PAHs-1 groups of HD, HS, and SH soils on the 56th day. The removal of Chr and 9-ClAnt became much lower, which were in the range of 42–47%, revealing the higher potential environmental risk.

#### 3.3.1. Effect of Native Microorganisms on the Degradation of PAHs

Considering the degradation effects of microbial on soil PAHs [55], the soils were unplanted and kept in the dark with water content around the soil field capacity to further clarify the contribution of native microorganisms to the degradation of PAHs. The removal of PAHs in the blank control groups was mainly due to leaching and abiotic loss, such as adsorption and volatilization [27]. The four PAHs showed some degradation, among which the degradation rate decreased with increasing initial PAH concentration (Figure S10). Generally, 9-ClAnt showed the lowest degradation regardless of the type of soil and its initial concentration. It is believed that the degradation of PAHs in this experimental group of soil mainly comes from the role of microorganisms. As seen in Figure S10, the concentration of the four PAHs decreased over time in all soils. Obvious removal of Ant and BaP was observed, especially in PAHs-1 and PAHs-2 groups. The degradation percentage of Ant were 81%, 76%, and 87% in PAHs-1 groups of HD, HS, and SH, respectively. Thus, soil microorganisms could contribute greatly to soil PAH degradation.

#### 3.3.2. Effect of Plants on the Degradation of PAHs

**Figure 4:**
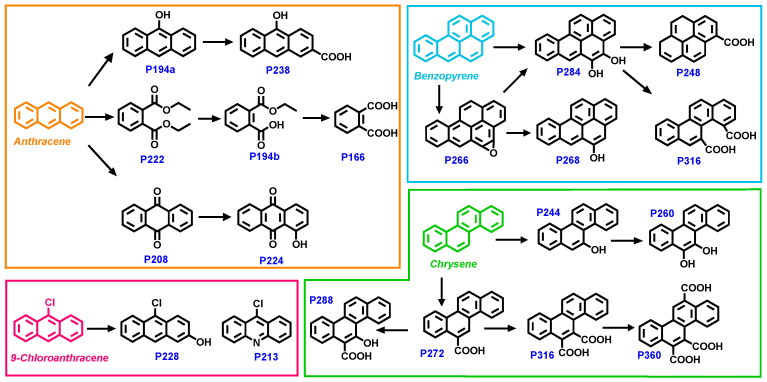
Proposed degradation pathways of the four PAHs. (Note that the intermediate compounds shown in the figure might have isomers.).

Further, the unplanted soils with sterilization were compared to clarify the influence of plantation (Figure S11). Compared with the unplanted group, the degradation of Chr in different soils was enhanced (Figure 4). When not planted, the removal efficiency percentages of Chr in the PAHs-1 experimental groups of Handan, Hengshui, and Shanghai soils on the 56th day were 13.5%, 20.7%, and 23.4%, respectively. In the case of ryegrass cultivation, the removal percentages were 66.0%, 42.4%, and 77.5%, respectively.

In addition, the degradation of 9-ClAnt was relatively low and not affected much by plant cultivation. With or without the planting of ryegrass, the removal percentages of 9-ClAnt in PAHs-1 groups of Handan, Hengshui, and Shanghai soils were 38.9% and 12.1%, 18.8% and 20.6%, 32.7%, and 36.4%, respectively. As illustrated in Section 3.1.2, the Cl-PAHs usually showed poor bioavailability, and their migration to plants was difficult. Moreover, Kamiya et al. examined the photolysis process of Cl-PAHs and found that Cl-PAHs were more stable than the corresponding parent PAHs [56]. Therefore, 9-ClAnt showed poor degradation, which was hardly affected by the vegetation of ryegrass.

To sum up, the vegetation of plants could facilitate the degradation of PAHs to some degree. In this study, the removal of Chr was improved by planting ryegrass. However, Cl-PAHs, such as 9-ClAnt, are relatively more difficult to degrade in natural environments, thus possessing higher environmental risks.

### 3.4. The Intermediate Products of PAHs

PAHs might be converted into other products under the combined action of plants, microorganisms, and other factors in the natural environment. The identification of intermediate products plays an important role in the environmental risk analysis of PAHs. In this study, soil and ryegrass shoot samples collected on day 56 were analyzed with HPLC/MS and GC/MS methods to identify the polar products and weak/non-polar products, respectively. A total of 19 intermediates of the four PAHs were identified. Detailed mass information on HPLC/MS analysis is shown in Table S10.

As shown in Figure 4, a hydroxylated product of ANT (anthracen-9-ol, P194a) was first generated, experiencing further carboxylation to form (10-hydroxyanthracene-2-carboxylic acid, P238). The formation of P222 (diethyl phthalate) was also observed under the effects of soil bacteria [57], which could be transformed into P194b (2-(ethoxycarbonyl)benzoic acid) and P166 (phthalic acid). Quinones are common conversion products of anthracene in the natural degradation process, which was confirmed in previous studies [58]. In addition, fungi could produce enzymes such as manganese peroxidase and laccases to convert PAHs into diphenol intermediates that are eventually oxidized to quinones [59]. In this work, anthracene-9,10-dione (P208) was identified, which could be transformed into P224 through hydroxylation.

In the degradation process of BaP, P266 was recognized through GC/MS analysis. The fungal community in the soil could produce certain intracellular enzymes, such as cytochrome P450 monooxygenase and epoxide hydrolase, which play a role in the degradation of 2–5-ring PAHs, resulting in the formation of epoxy products [60,61]. Moreover, bacteria could use BaP as a carbon source or synthesize enzymes that could transform the five-ring PAHs [10,62] Afterwards, P266 could be further transformed into the mono-hydroxylation product P268 or the di-hydroxylation product P284. Finally, P248 and P316, which were both identified by HPLC/MS, were formed from P284 after the fission process [63,64].

Continuous hydroxylation of Chr in soil could lead to hydroxylation products (P244 and P260) in sequence. Meanwhile, P272, P316, and P360, as monocarboxylic, dicarboxylic, and tricarboxylic products, respectively, were formed from sequential carboxylation processes of Chr. Then, P244 and P272 were converted to the product P288 through carboxylation and hydroxylation, respectively. As for 9-ClAnt, its hydroxylation product P228 could be identified by HPLC/MS, and the nitrogenated product P213 was found in the GC/MS analysis.

In summary, the four PAHs could undergo hydroxylation reactions to form corresponding hydroxyl addition products. For the three unchlorinated PAHs (Ant, BaP, and Chr), carboxylated products could be formed through carboxylation (e.g., P238, P272, P288, P316, and P360) or through ring opening (e.g., P166 and P316). In addition to microbial density and microbial diversity, soil properties like texture, permeability, moisture content, and organic matter content are also important factors affecting PAH degradation in soil [65]. Therefore, it is necessary to clarify the intermediate products of PAHs during natural degradation at contaminated sites as well as their potential impact on indigenous microorganisms, which are of great significance for environmental risk analysis related to PAHs.

## 4. Conclusions

This study examined the effects of ryegrass planting and soil native microbial communities on the degradation of PAH mixture in three types of soils, aiming to elucidate the interaction of PAHs with microbial communities and plants. The plant height was the lowest in PAH-contaminated Hengshui soil, and it took the longest time (about 5–6 days) for seed germination. Under PAH stress, the species abundance of some PAHs-degrading bacteria, such as *Pseudomonas*, *Bacillus*, *Sphingomonas*, and *Lysobacter*, changed in the soil, but the changes were not always positively correlated with the exposure concentration of PAHs. From the comparison of the degradation of PAHs in soils with and without planting of ryegrass, it was found that microorganisms in soils played an important role in the degradation of PAHs, while the plantation of ryegrass improved the degradation efficiency of chrysene. During the natural degradation process, 19 intermediates were identified by combined use of HPLC/MS and GC/MS, and hydroxylation and carboxylation were the common reaction pathways. This study is of great significance in clarifying the potential impact of PAHs on the primary microbial environment in contaminated sites and can provide valuable information for assessing the potential risks of PAHs in natural degradation processes.

## Figures and Tables

**Table 1:** Groups names, treatment of different experimental groups, and data utilization in different sections.

Group Name	Vegetation	Sterilization	Darkness	PAH Load
SH * _BK	√	×	×	×
SH_P1 (P2,P3)	√	×	×	√
SH_B1 (B2,B3)	×	×	√	√
SH_C1 (C2,C3)	×	√	×	√
Data utilization in different sections				
Group Name	Plant Height Analysis	Microbial Diversity		Degradation Results
SH * _BK	√	√		×
SH_P1 (P2,P3)	√	√		√
SH_B1 (B2,B3)	×	×		√
SH_C1 (C2,C3)	×	×		√

* Note: Taking the naming of soil samples from Shanghai (SH) as an example, the experimental groups set up in Handan (HD) and Hengshui (HS) were the same; numbers 1–3 represent the concentration gradients from 250 mg/kg, 500 mg/kg, and 1000 mg/kg.

## Data Availability

The data presented in this study are available on request from the corresponding author.

## Supplementary Materials

The following supporting information can be downloaded at: https://www.mdpi.com/article/10.3390/toxics12050361/s1, Text S1: Detailed information of chemicals and reagents; Text S2: Pollution of PAHs in the soil of the three industrial parks; Text S3: Detailed information of the pot experiments; Text S4: Detailed process of HPLC, HPLC-MS, and GC-MS analyses; Text S5: Detailed process of the sequencing process; Table S1: Physicochemical properties of the soil samples used in the experiment; Table S2: The concentration gradients of PAHs; Table S3: The initial concentration of PAHs after artificial contamination (mg/kg); Table S4: HPLC detection methods for the PAHs used in the study; Table S5: Contents of four PAHs in ryegrass after 56 days of growth; Table S5: The effectiveness of sequencing results; Table S6: The effectiveness of sequencing results of the vegetation groups; Table S7: Alpha diversity indices of bacteria and fungi of different soil samples; Table S8: Statistical table of species taxonomic annotation results; Table S9: The relative abundance of fungal communities in Shanghai, Handan, and Hengshui soils with different PAH concentrations; Table S10: Accurate mass measurements of the degradation products of four PAHs as determined by HPLC-TOF-MS/MS; Figure S1: Location of the three industrial parks in China; Figure S2: Sampling sites in Shanghai (a), Handan (b), and Hengshui (c); Figure S3: The standard curve of peak area and solution concentration for Ant (a), 9-ClAnt (b), BaP (c), and Chr (d).; Figure S4: The rank abundance curve of bacteria in Shanghai (a) and Handan (b) soil samples; Figure S5: PCoA profile of community dissimilarity indices (Bray−Curtis) calculated from the OUT tables of different soils; Figure S6: Relative abundance of bacteria at the phylum level in Shanghai (a), Handan (b), and Hengshui (c) soils with different concentrations of PAHs; Figure S7: Comparative heat map of bacteria in Handan soil samples; Figure S8: Comparative heat map of bacteria in Shanghai soil samples; Figure S9: Comparative heat map of bacteria in Hengshui soil samples; Figure S10: The degradation of PAHs in Handan (a–c), Hengshui (d–f), and Shanghai (g–i) soils in the dark with no planting of ryegrass; Figure S11: The loss of PAHs in Handan (a–c), Hengshui (d–f), and Shanghai (g–i) soils in the dark with sterilization and no planting of ryegrass [57,65,66,67,68,69,70].
