# Supplementary material for: Transformation and Degradation of PAH Mixture in Contaminated Sites: Clarifying Their Interactions with Native Soil Organisms
Source: Toxics. 2024 May 13;12(5):361. doi: 10.3390/toxics12050361 (PMC11126024; doi:10.3390/toxics12050361)
Supplement: Supplementary file 1 [file toxics-12-00361-s001.zip › toxics-2994432-supplementary.pdf]

Supporting Information

Transformation and Degradation of PAHs Mixture in Contaminated Sites: Clarifying Their Interactions with Native Soil Organisms

Xiaoyu Li[1], Shengnan Zhang[1], Ruixue Guo[1], Xuejing Xiao[1], Boying Liu[1], Rehab Khaled. Mahmoud [2], Mostafa R. Abukhadra[3], Ruijuan Qu[1], and Zunyao, Wang[1,*]

1. State Key Laboratory of Pollution Control and Resources Reuse, School of the Environment, Nanjing University, Nanjing 210023, Jiangsu, PR China; quruijuan0404@nju.edu.cn (R.Q.); wangzy@nju.edu.cn (Z.W.).

2. Faculty of science, Beni Suef University, Beni-Suef, Egypt; rehabkhaled@science.bsu.edu.eg.

3. Materials Technologies and their applications Lab, Faculty of Science, Beni-Suef University, Beni Suef city, Egypt; abukhadra89@science.bsu.edu.eg.

Text S1 Detailed information of chemicals and reagents.

Ant (purity ⩾ 98%), 9-ClAnt (purity ⩾ 97%), Chr (purity ⩾ 97%) and BaP (purity ⩾ 98%) were obtained from Macklin Co., Ltd. (Shanghai, China). The stock solutions of PAHs were prepared at a concentration of 1.0 g/L in acetone and stored at 4 °C in the dark. Ultrapure water was generated from a Milli-Q water purification system (18.25 MΩ·cm, Millipore, Bedford, USA). Chromatographic grade n-hexane, acetone, and methanol were supplied by Merck (Darmstadt, Germany).

Text S2 Pollution of PAHs in the soil of the three industrial parks.

The sampling points from the three industrial parks all showed varying degrees of PAHs pollution. The concentrations of 16 USEPA priority PAHs were measured by EPA 8270E and EPA 3545A standard methods. The total concentrations of 12 PAHs (Phenanthrene, Anthracene, Fluoranthene, Pyrene, 1,2-Benzanthracene, Chrysene, Benzo(b)fluoranthene, Benzo(k)fluoranthene, Benzo(a)pyrene, Indeno(1.2.3-c,d)pyrene, Dibenz(a,h)anthracene, Benzo(g,h,i)perylene) were measured to be 39 mg/kg and 7 mg/kg in SH and HD soil , respectively. By contrast, the total concentration of 16 priority PAHs in the soil samples collected from HS soil was relatively low, while benzo(a)pyrene (700 μg/kg) was found to exceed the Risk Control Standard for Soil Contamination of Development Land (DB13/T 5216-2020).

Text S3. Detailed information of the pot experiments.

After stones and large plant residuals were removed, the collected soils were air-dried, and ground to 60 mesh particles for use. Different volumes of PAHs stock solutions were added to the soil samples, which were placed in dark in the fume hood to allow for natural evaporation of organic solvent. 500 g PAHs-contaminated soils were packed into each of the plastic pots (15.5 cm diameter × 15.0 cm height). Soils were watered and equilibrated for 7 days before sowing seeds of ryegrass which were purchased from Jiangsu Leerda Seed Industry Co., LTD (Jiangsu, China). Whole seeds of the same size were selected, sterilized in a 3% hydrogen peroxide solution for 20 min, and then washed three times with water. After natural air drying, the seeds were used for sowing, and 5 seedlings with good growth were reserved in each pot. The experimental period lasted from September to November of 2022 in a greenhouse at Nanjing University (Nanjing, China, 32.12°N, 118.96°E), under natural light irradiation. The pot experiments were conducted from September to November. During the pot experiments, soil moisture was kept at 60% of field water capacity, and plant height was recorded on the first day and every two days thereafter. The pots were placed in a culture room at a constant temperature of 25 ± 2°C, and the positions of the pots were randomly changed every two days. On the 7th, 14th, 28th, 42nd, and 56th day since sowing, a soil column (1 cm diameter, 10 cm height) was collected from each pot for quantitative analysis of PAHs and identification of transformation products.

Text S4 Detailed process of HPLC, HPLC-MS, and GC-MS analysis.

HPLC analysis

Quantification of PAHs was performed on an HPLC instrument (Flexar, PerkinElmer, USA) equipped with an L-2300 pump and an L-2420 UV–vis detector. Separation of components was conducted through a Zorbax SB-C18 column (4.6 × 150 mm, 5 μm) with the column temperature maintained at 30 °C. Other information for the test conditions of the four PAHs were listed in Table S4. The standard curve of peak area and solution concentration could be seen in Figure S3.

HPLC-MS analysis

The polar products were identified by an Agilent 1260 Infinity HPLC system coupled with a high-resolution quadrupole time-of-flight mass spectrometer (Triple TOF 5600, AB Sciex, USA), which was operated with an electrospray ionization (ESI) source in negative mode to record the full scanning spectrum (m/z 50–1500). Ten microliter samples were eluted on a Thermo BDS Hypersil C18 column (100 × 2.1 mm, 2.4 μm particle size) for separation. The mobile phase consisting of 0.1% formic acid in water (A) and methanol (B) was eluted at a flow rate of 0.2 mL min^{-1}. The gradient elution program was performed according to our previous study [1]. It started at 90% A for 2 min, decreased to 10% A in 1 min and kept for 23 min, then returned to the starting condition in 1 min and equilibrated for 8 min.

GC-MS analysis

The products with weak or even no polarity were identified by a TSQ Quantum gas chromatograph-mass spectrometer (GC-MS, Thermo Scientific, USA) equipped with an electron ionization (EI) source. Two-microliter samples were injected into a DB-5MS capillary column (30 m

length × 0.25 μm film thickness, 320 μm i.d., J&K Scientific, USA) under splitless mode. Nitrogen (purity > 99.999%) was used as the carrier gas, and the flow rate was 1.2 mL min^{-1}. The temperatures of injector, ion source and transfer line were 280 °C, 250 °C and 280 °C, respectively. The column temperature was initially held at 80 °C for 1 min, increased at 25 °C min^{-1} to 200 °C and maintained for 2 min, then ramped at 1 °C min^{-1} to 213 °C and kept for 1 min. Full scanning MS spectrum (m/z 50–500) was recorded.

Text S5 Detailed process of the sequencing process.

DNA Extraction

Total genomic DNA samples were extracted using the OMEGA Soil DNA Kit (M5635-02) (Omega Bio-Tek, Norcross, GA, USA), following the manufacturer's instructions, and stored at -20 °C prior to further analysis. The quantity and quality of extracted DNAs were measured using a NanoDrop NC2000 spectrophotometer (Thermo Fisher Scientific, Waltham, MA, USA) and agarose gel electrophoresis, respectively.

16S rRNA Gene Amplicon Sequencing

PCR amplification of the bacterial 16S rRNA genes V3–V4 region was performed using the forward primer 338F (5'-ACTCCTACGGGAGGCAGCA-3') and the reverse primer 806R (5'-GGACTACHVGGGTWTCTAAT-3'). Sample-specific 7-bp barcodes were incorporated into the primers for multiplex sequencing. The PCR components contained 5 µL of buffer (5×), 0.25 µl of Fast pfu DNA Polymerase (5U/µL), 2 µl (2.5 mM) of dNTPs, 1 µL (10 uM) of each Forward and Reverse primer, 1 µL of DNA Template, and 14.75 µL of ddH_2O. Thermal cycling consisted of initial denaturation at 98 °C for 5 min, followed by 25 cycles consisting of denaturation at 98 °C for 30 s, annealing at 53 °C for 30 s, and extension at 72 °C for 45 s, with a final extension of 5 min at 72 °C. PCR amplicons were purified with Vazyme VAHTSTM DNA Clean Beads (Vazyme, Nanjing, China) and quantified using the Quant-iT PicoGreen dsDNA Assay Kit (Invitrogen, Carlsbad, CA, USA). After the individual quantification step, amplicons were pooled in equal amounts, and pair-end 2×250 bp sequencing was performed using the Illlumina NovaSeqplatform with NovaSeq 6000 SP Reagent Kit (500 cycles).

Sequence Analysis

Microbiome bioinformatics were performed with QIIME2 2019.4 [2] with slight modifications according to the official tutorials (https://docs.qiime2.org/2019.4/tutorials/). Briefly, raw sequence data were demultiplexed using the demux plugin followed by primers cutting with cutadapt plugin [3]. Sequences were then quality filtered, denoised, merged and chimera removed using the DADA2 plugin [4]. Non-singleton amplicon sequence variants (ASVs) were aligned with mafft [5] and used to construct a phylogeny with fasttree2 [6].

Table S1 Physicochemical properties of the soil samples used in the experiment.

Area	Soil type	Soil texture (%, mm)			pH	Soil organic carbon	Cation exchange capacity
		<0.002	0.05-0.002	2-0.05		(SOC, g/kg)	(CEC, cmol/kg)
Handan	Sandy loam	6.85	34.41	58.74	8.06	12.47	13.55
Hengshui	Loam	12.56	41.71	45.73	7.55	21.32	9.82
Shanghai	Silt Loam	17.36	50.73	31.91	8.10	22.38	10.24

Table S2 The concentration gradients of PAHs.

PAHs (mg/kg)	Chr	Ant	9-ClAnt	BaP	Sum.
PAHs-1	25	5	5	15	50
PAHs-2	125	25	25	75	250
PAHs-3	500	100	100	300	1000

Table S3 The initial concentration of PAHs after artificial contamination (mg/kg).

	HD_1	HD_2	HD_3	HS_1	HS_2	HS_3	SH_1	SH_2	SH_3
Ant	2.51	19.69	84.47	4.00	20.08	96.33	4.71	24.46	93.80
9-Ant	4.56	24.10	88.34	6.23	28.44	105.37	4.02	25.13	98.01
BaP	11.61	79.70	273.40	9.40	60.10	284.12	15.86	68.99	320.17
Chr	10.69	115.52	477.02	9.52	111.07	511.32	16.99	118.43	452.33

Table S4 HPLC detection methods for the PAHs used in the study.

PAHs	Detection wavelength (nm)	Flow rate ($mLmin^{-1}$)	Mobile phase	
			Acetonitrile	Aqueous formic acid
Chr	268	1.35	20	80
Ant	253	1.35	15	85
9-ClAnt	265	1.35	15	85
BaP	290	1.35	5	95

Table S5 Contents of 4 PAHs in ryegrass after 56 days of growth.

Samples (μg/kg)		Ant	BaP	Chr	9-ClAnt
Handan (HD)	PAHs-P1	0.36±0.06	0.24±0.03	0.59±0.14	0.61±0.10
	PAHs-P2	0.72±0.14	0.80±0.11	2.04±0.24	0.38±0.08
	PAHs-P3	1.14±0.32	1.17±0.21	2.18±0.37	0.32±0.11
Shanghai (SH)	PAHs-P1	0.47±0.09	0.45±0.10	2.13±0.24	0.32±0.03
	PAHs-P2	0.52±0.11	0.73±0.24	4.68±0.08	0.88±0.10
	PAHs-P3	0.57±0.05	0.86±0.19	6.34±0.35	0.46±0.12

Table S6 The effectiveness of sequencing results of the vegetation groups.

SampleID	Input	Non-chimeric	Validity (%)
SH_BK	133258	122070	91.6
SH_P1	135489	122198	90.2
SH_P2	137111	124575	90.9
SH_P3	130533	119058	91.2
HD_BK	164475	152058	92.5
HD_P1	83826	74406	88.8
HD_P2	97664	89936	92.1
HD_P3	77185	69617	90.2
HS_BK	69493	63011	90.1
HS_P1	98772	89713	90.1
HS_P2	114729	103677	90.4
HS_P3	72875	67533	92.7

Table S7 Alpha diversity indices of bacteria and fungi of different soil samples.

Sample	Number	SH_BK	SH_P1	SH_P2	SH_P3	HD_BK	HD_P1	HD_P2	HD_P3	HS_BK	HS_P1	HS_P2	HS_P3
Bacteria	Chao1	418.8	970.2	554.0	530.2	6035.1	1552.0	1347.9	1320.6	475.3	39.3	58.7	27.5
	Simpson	0.956	0.932	0.981	0.974	0.995	0.993	0.984	0.988	0.969	0.925	0.985	0.922
	Shannon	6.101	6.358	7.021	6.836	10.225	8.673	8.016	8.144	6.153	3.893	6.724	3.864
	Observed species	418	969	553	528	5404	1545	1302	1316	474	37	48	27
	Coverage	1.00	1.00	1.00	1.00	0.98	0.99	0.98	0.99	1.00	1.00	0.99	1.00
Fungi	Chao1	95.0	105.3	108.9	101.1	134.0	8.0	17.1	44.0	24.0	29.1	25.0	8.0
	Simpson	0.535	0.834	0.809	0.846	0.720	0.605	0.560	0.866	0.613	0.127	0.065	0.018
	Shannon	2.601	3.332	3.179	3.302	3.652	1.671	1.617	3.223	1.847	0.512	0.310	0.094
	Observed species	95	105	109	101	134	8	17	44	24	29	25	8
	Coverage	0.99	0.99	0.99	0.99	1.00	1.00	0.99	0.99	0.99	0.99	0.99	0.99

Table S8 Statistical table of species taxonomic annotation results.

	Bacteria					Fungi				
Sample	Phylum	Class	Order	Family	Genus	Phylum	Class	Order	Family	Genus
SH_BK	16	32	63	91	117	2	9	14	17	20
SH_P1	21	38	78	130	201	2	9	13	15	22
SH_P2	16	27	63	94	141	4	10	14	19	26
SH_P3	15	33	63	95	140	3	9	14	17	19
HD_BK	19	54	111	174	269	3	10	20	31	46
HD_P1	20	56	107	166	243	1	2	3	3	2
HD_P2	18	40	81	127	185	3	7	15	22	23
HD_P3	19	45	94	144	222	1	3	5	5	4
HS_BK	8	16	30	43	48	1	3	6	7	8
HS_P1	6	8	12	12	10	2	6	9	11	10
HS_P2	8	19	36	45	59	1	2	3	3	3
HS_P3	3	7	11	14	11	3	6	9	9	7

Table S9 The relative abundance of fungal communities in Shanghai, Handan and Henshui soils with different PAHs concentrations.

Sample	Relative abundance (%)							
	Ascomycota	Basidiomycota	Mucoromycota	Mortierellomycota	Rozellomycota	Blastocladiomycota	Mortierellomycota	Others
SH_BK	10.8	2.8	/	/	/	/	/	86.4
SH_P1	53.0	0.2	/	/	/	/	/	46.8
SH_P2	26.1	*	*	/	*	/	/	73.8
SH_P3	16.7	0.2	*	/	/	/	/	83.0
HD_BK	91.0	2.8	/	/	/	/	2.7	3.5
HD_P1	99.9	/	/	/	/	/	/	0.1
HD_P2	43.8	*	/	/	/	/	*	56.1
HD_P3	89.2	/	/	/	/	/	/	10.8
HS_BK	99.9	/	/	/	/	/	/	*
HS_P1	99.9	*	/	/	/	/	/	/
HS_P2	1	/	/	/	/	/	/	/
HS_P3	98.9	*	/	/	/	*	/	1.1

Note that * represents the relative abundance is lower than 0.01%, and / represents no detection.

Table S10. Accurate mass measurements of the degradation products of 4 PAHs as determined by HPLC-TOF-MS/MS.

Compound	Retention time ($R_{t,}$ min)	Molecular formula	Experimental mass (*m/z*)	Calculated mass (*m/z*)	Error (ppm)
P194(-)	8.127	$C_{14}H_{10}O$	193.0658	193.0672	5.69
P238(-)	12.46	$C_{15}H_{10}O_3$	237.0557	237.0554	-1.27
P224(-)	10.29	$C_{11}H_7O_5$	223.0400	223.0388	-5.38
P284(-)	11.53	$C_{20}H_{12}O_2$	283.0764	283.0743	-7.42
P248(-)	12.73	$C_{17}H_{12}O_2$	247.0764	247.0768	1.62
P260(-)	11.25	$C_{18}H_{12}O_2$	259.0764	259.0774	3.86
P288(-)	11.45	$C_{19}H_{12}O_3$	287.0713	287.0702	-3.82
P360(-)	13.02	$C_{21}H_{12}O_6$	359.1077	359.1067	-2.79

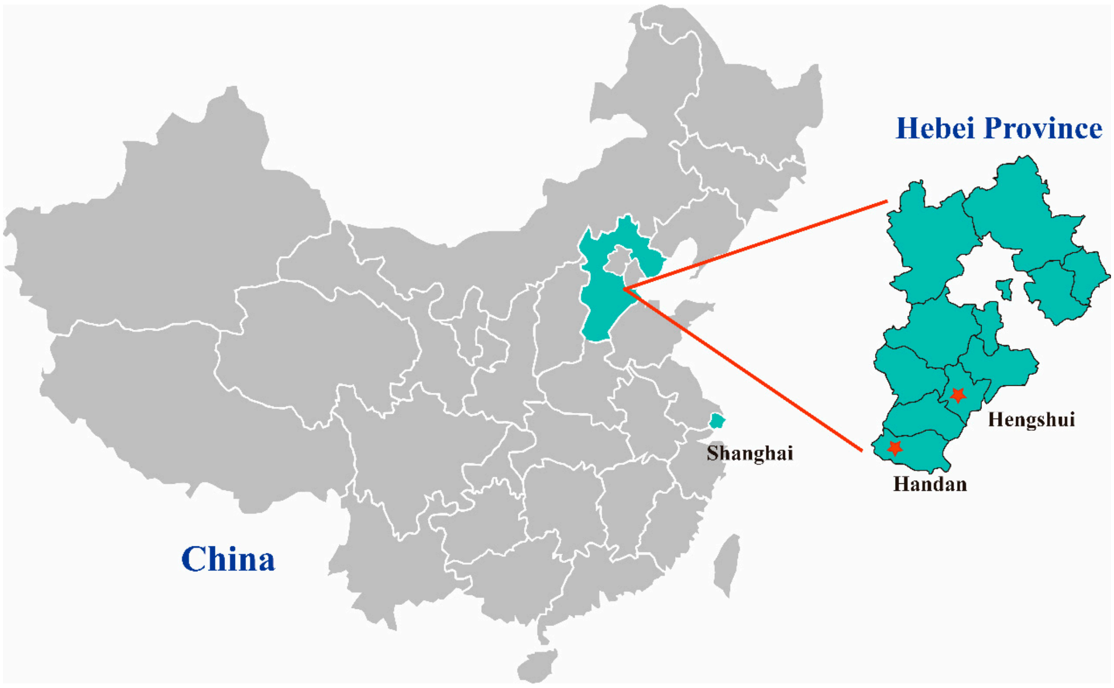

Figure S1 Location of the three industrial parks in China.

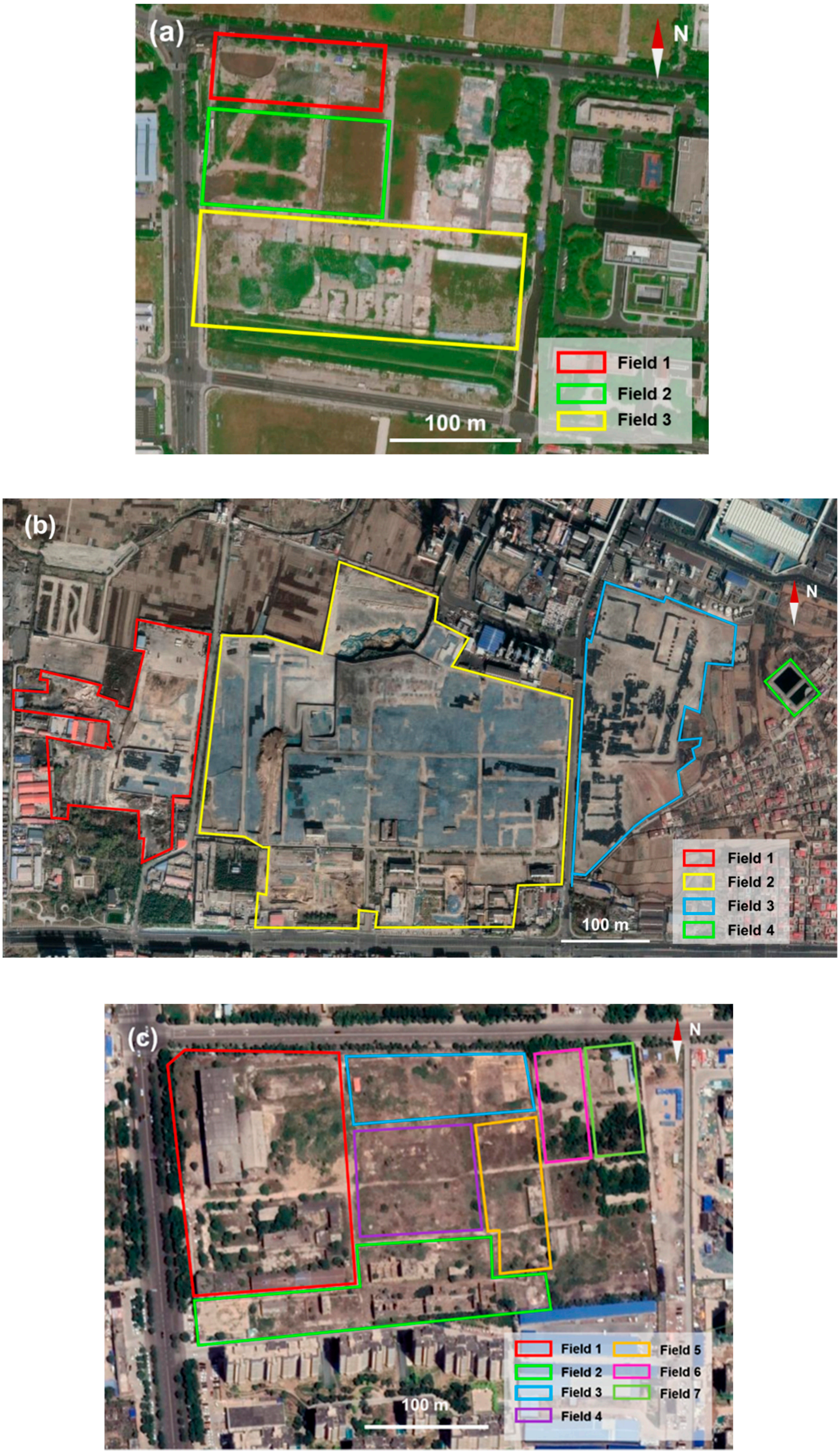

Figure S2 Sampling sites in Shanghai (a) Handan (b) and Hengshui (c).

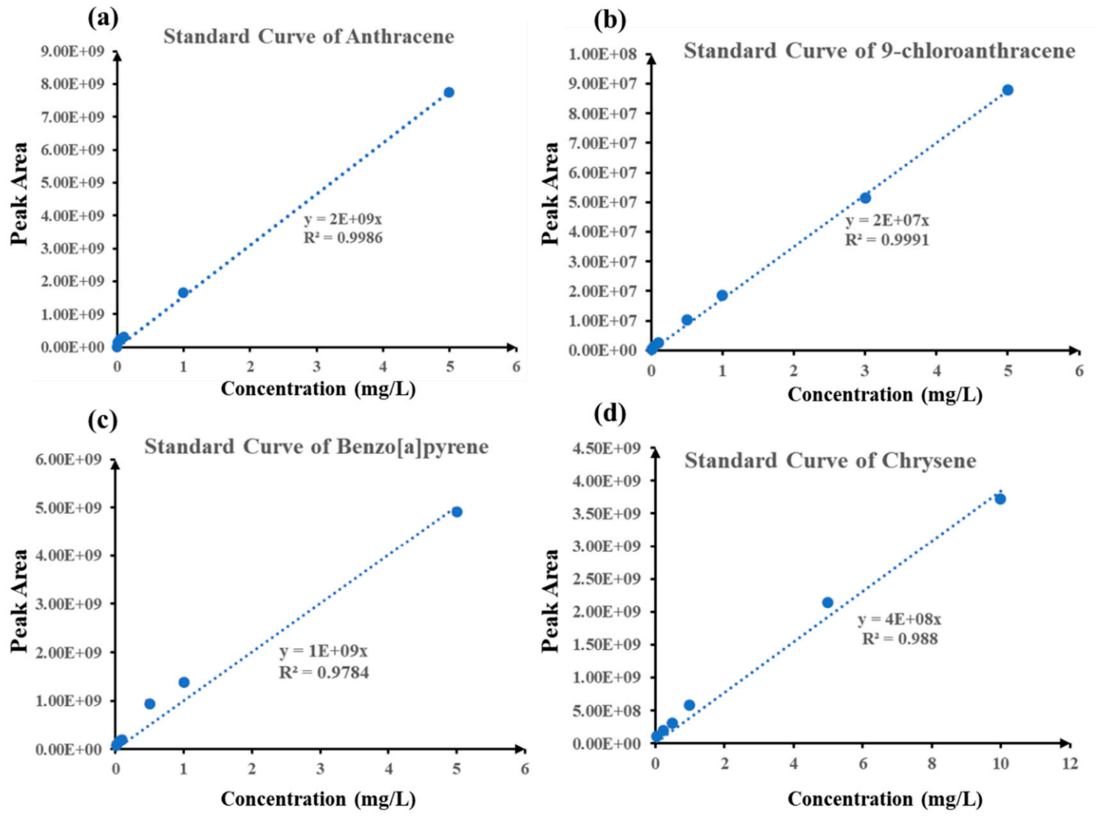

Figure S3 The standard curve of peak area and solution concentration for Ant (a), 9-ClAnt (b), BaP (c) and Chr (d).

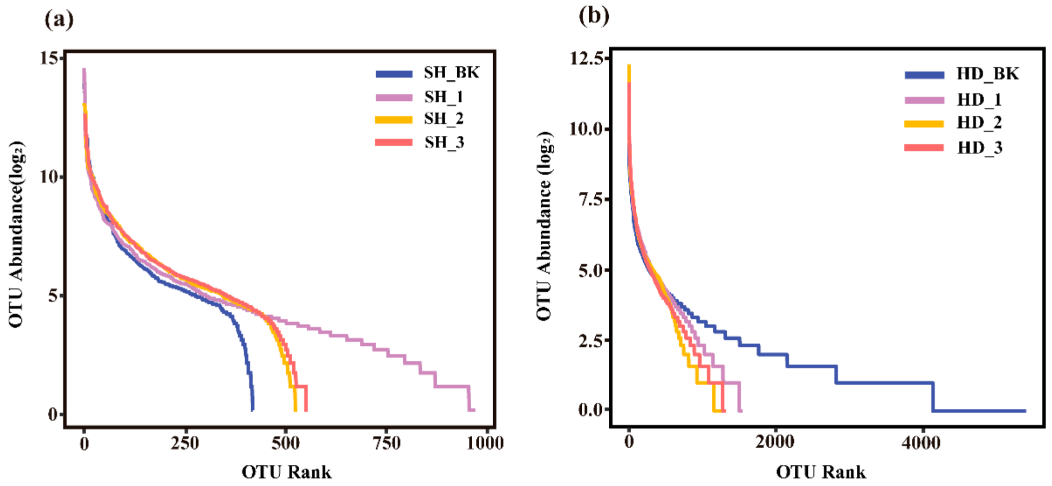

Figure S4 The rank abundance curve of bacteria in Shanghai (a) and Handan (b) soil samples.

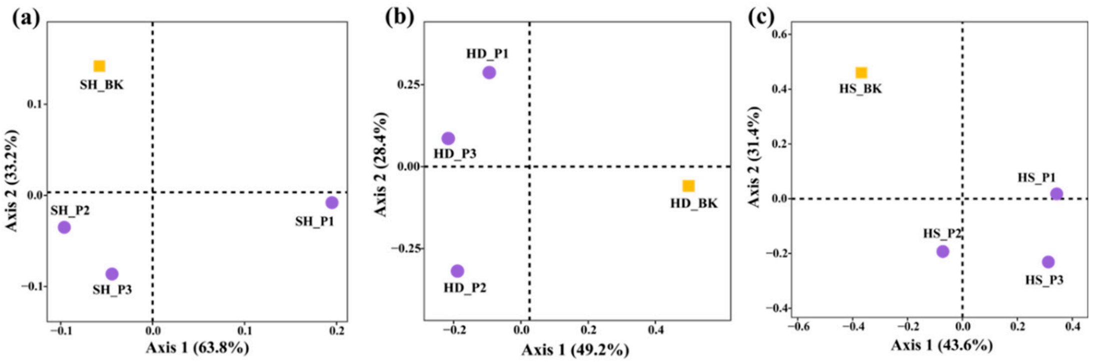

Figure S5 PCoA profile of community dissimilarity indices (Bray−Curtis) calculated from the OUT tables of different soils of Shanghai (a), Handan (b) and Hengshui (c). (BK: Control group without contamination, P1: 50 mg/kg, P2: 250 mg/kg; P3: 1000 mg/kg)

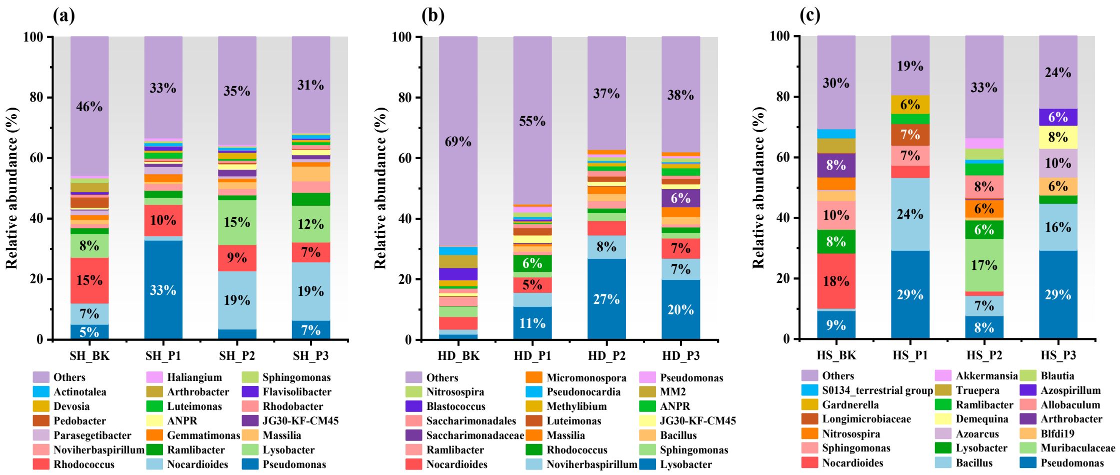

Figure S6 Relative abundance of bacteria at phylum level in in Shanghai (a), Handan (b) and Henshui (c) soils with different concentrations of PAHs.

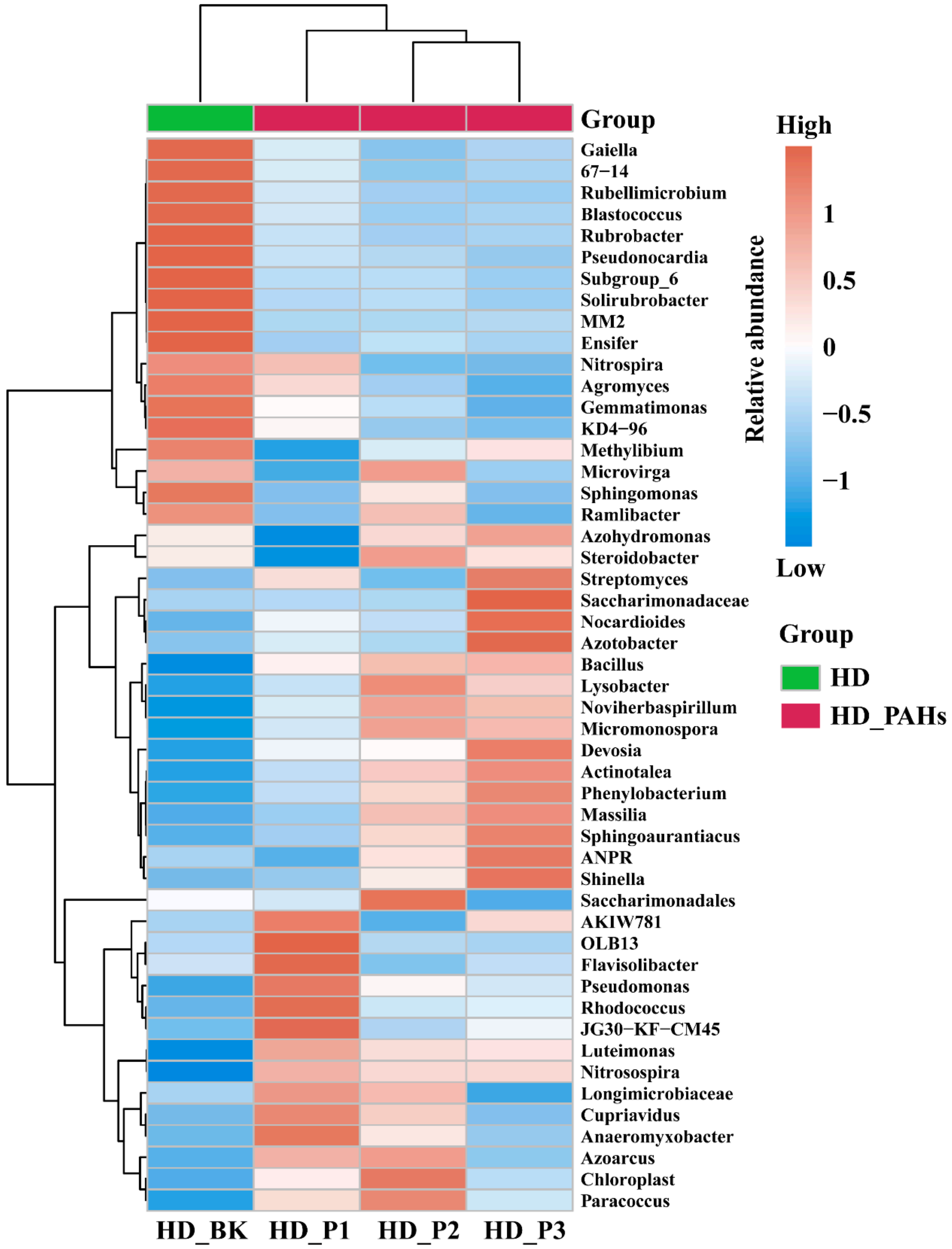

Figure S7 Comparative heatmap of bacteria in Handan soil samples. (The abundance data of the top 50 genera based on average abundance was used, the red blocks indicate that the abundance of the genus in this sample is higher compared to other samples while the blue blocks suggest that the abundance in this sample was lower than other samples.)

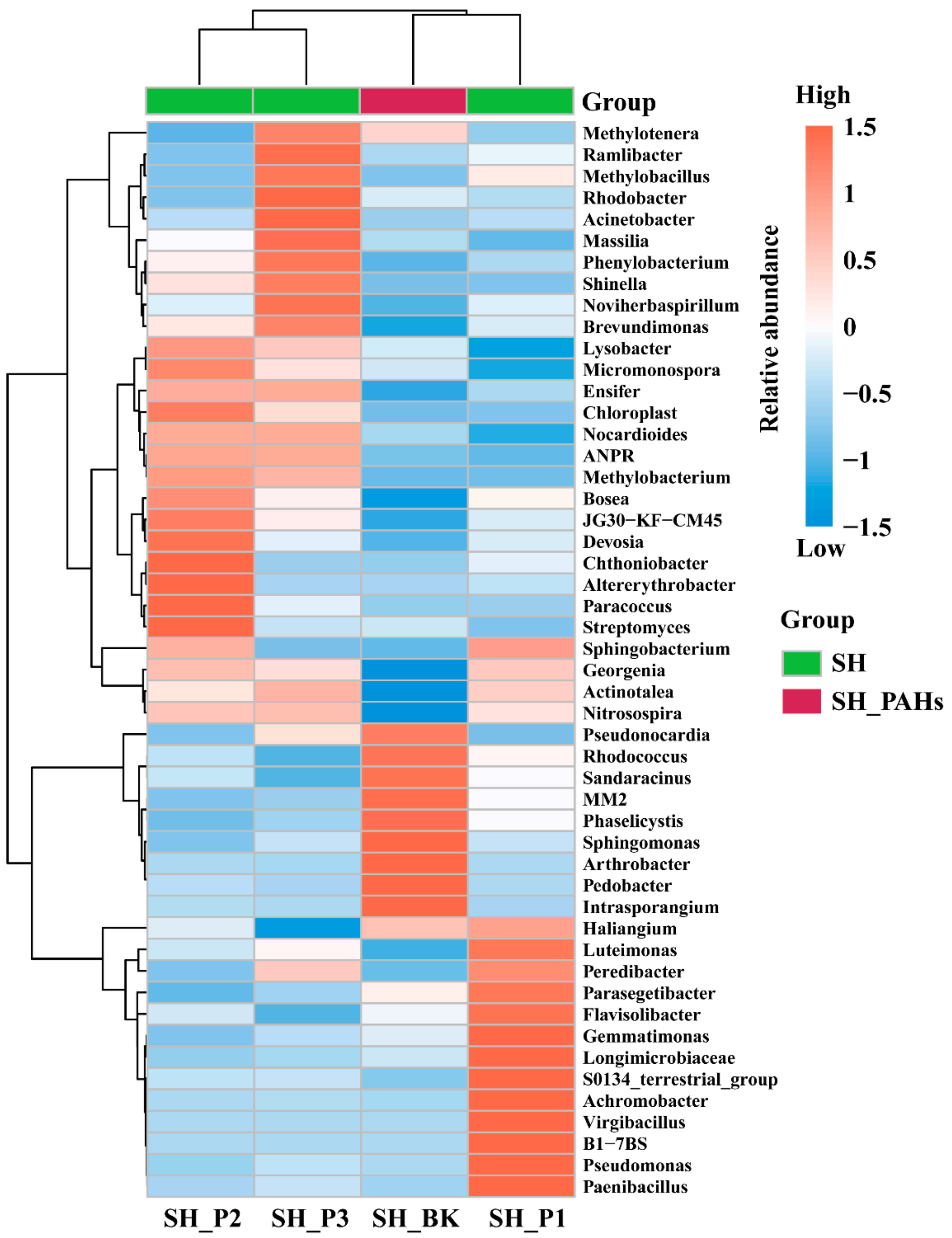

Figure S8 Comparative heat map of bacteria in Shanghai soil samples. (The abundance data of the top 50 genera based on average abundance was used, the red blocks indicate that the abundance of the genus in this sample is higher compared to other samples while the blue blocks suggest that the abundance in this sample was lower than other samples.)

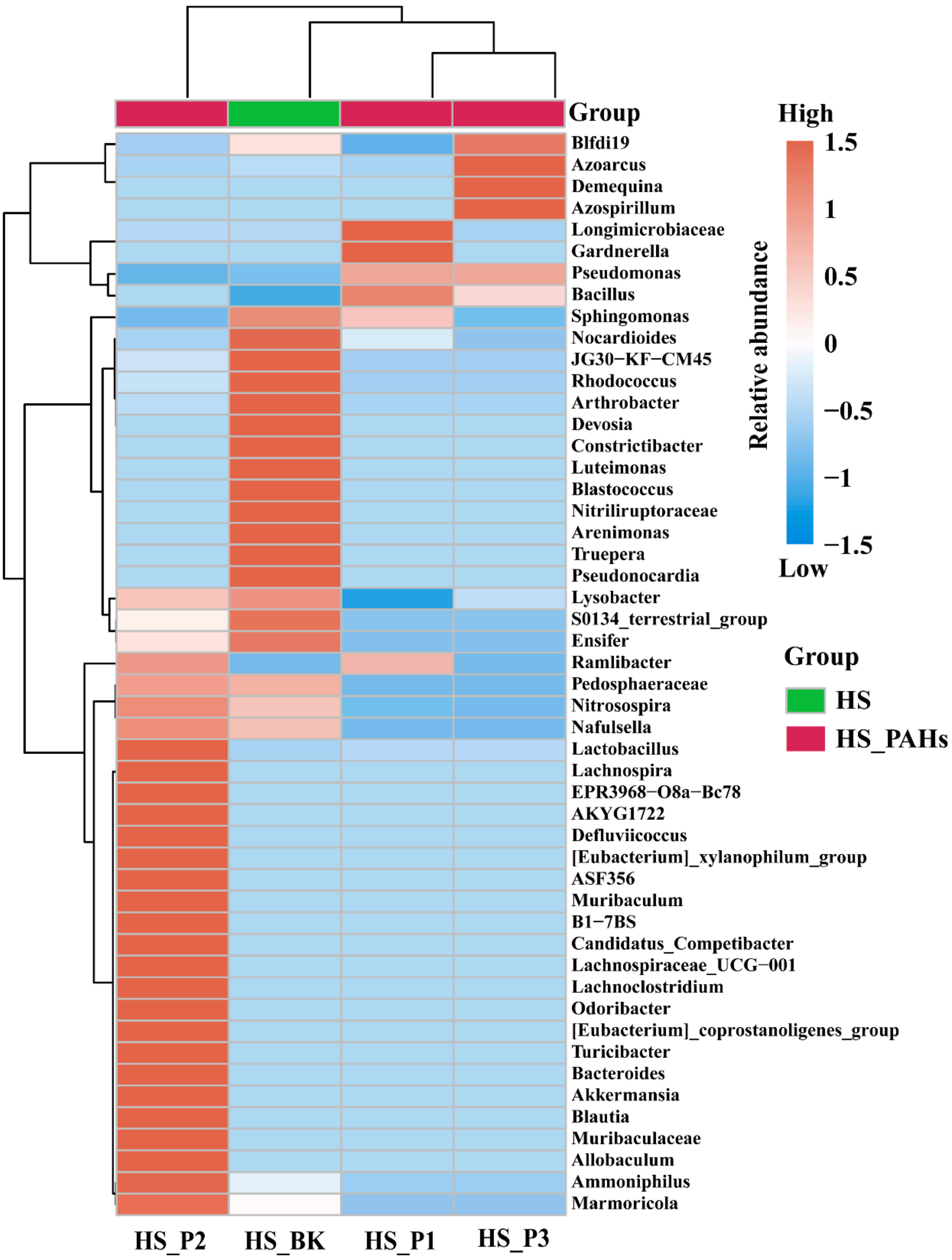

Figure S9 Comparative heatmap of bacteria in Hengshui soil samples. (The abundance data of the top 50 genera based on average abundance was used, the red blocks indicate that the abundance of the genus in this sample is higher compared to other samples while the blue blocks suggest that the abundance in this sample was lower than other samples.)

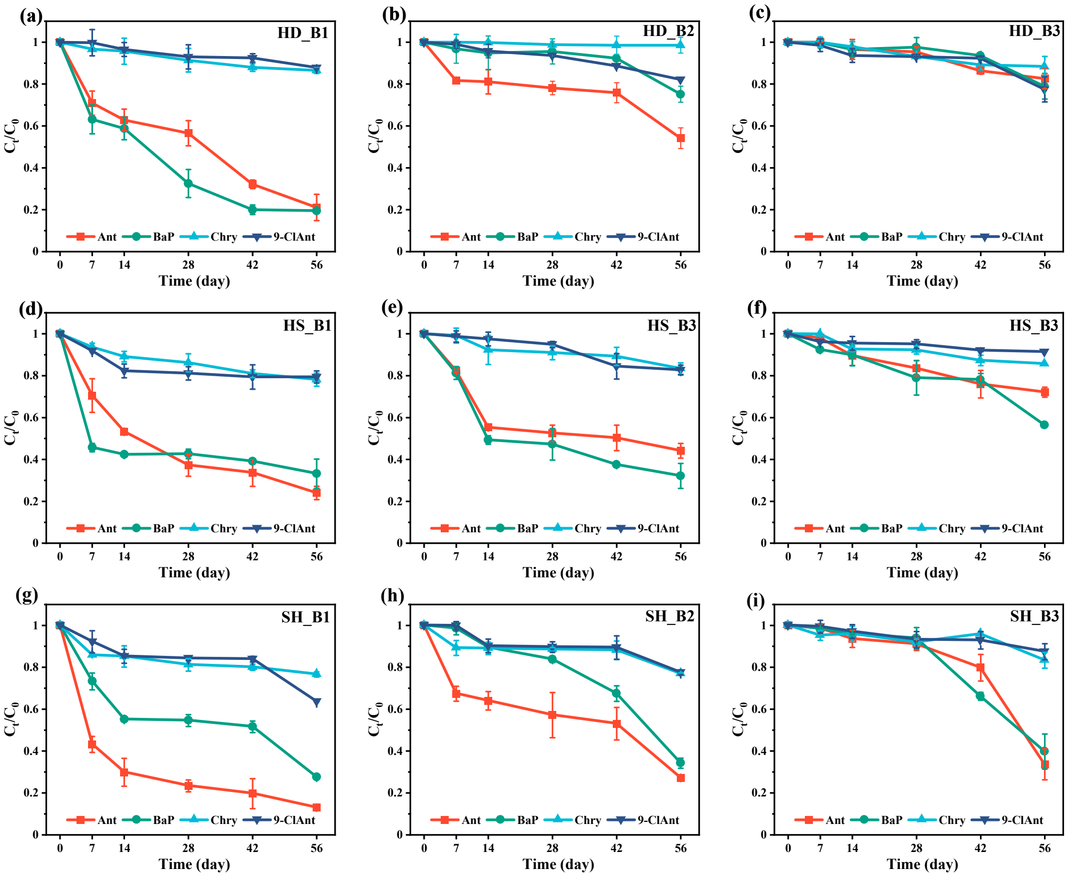

Figure S10 The degradation of PAHs in Handan (a-c), Hengshui (d-f) and Shanghai (g-i) soils in dark with no planting of ryegrass. (SH: Shanghai, HD: Handan, HS: Hengshui; C1: 50 mg/kg, C2: 250 mg/kg; C3: 1000 mg/kg)

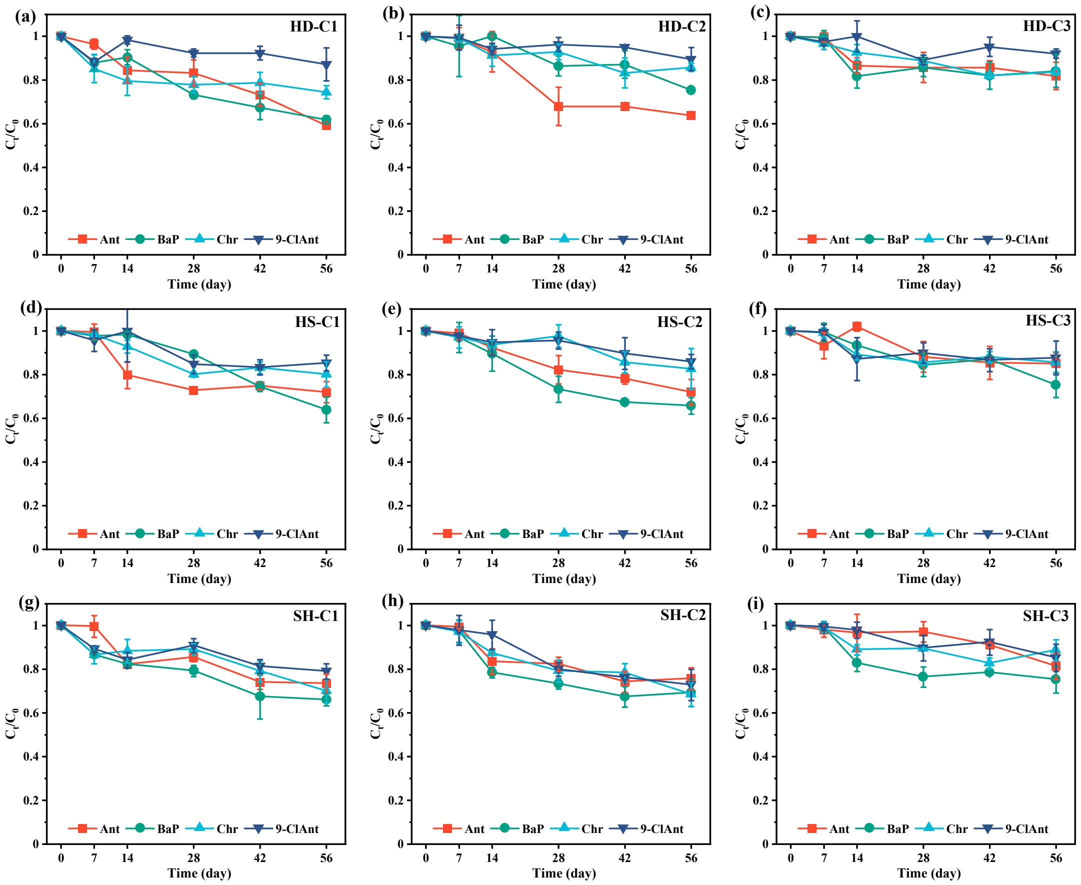

Figure S11 The loss of PAHs in Handan (a-c), Hengshui (d-f) and Shanghai (g-i) soils in dark with sterilization and no planting of ryegrass. (SH: Shanghai, HD: Handan, HS: Hengshui; C1: 50 mg/kg, C2: 250 mg/kg; C3: 1000 mg/kg)

Reference

1. Tu, Z., Qi, Y., Tang, X., Wang, Z. and Qu, R. 2023. Photochemical transformation of anthracene (ANT) in surface soil: Chlorination and hydroxylation. J Hazard Mater 452, 131252.

2. E. Bolyen, J.R. Rideout, M.R. Dillon, N.A. Bokulich, C.C. Abnet, G.A. Al-Ghalith, H. Alexander, E.J. Alm, M. Arumugam, F. Asnicar, Y. Bai, J.E. Bisanz, K. Bittinger, A. Brejnrod, C.J. Brislawn, C.T. Brown, B.J. Callahan, A.M. Caraballo-Rodríguez, J. Chase, E.K. Cope, R. Da Silva, C. Diener, P.C. Dorrestein, G.M. Douglas, D.M. Durall, C. Duvallet, C.F. Edwardson, M. Ernst, M. Estaki, J. Fouquier, J.M. Gauglitz, S.M. Gibbons, D.L. Gibson, A. Gonzalez, K. Gorlick, J. Guo, B. Hillmann, S. Holmes, H. Holste, C. Huttenhower, G.A. Huttley, S. Janssen, A.K. Jarmusch, L. Jiang, B.D. Kaehler, K.B. Kang, C.R. Keefe, P. Keim, S.T. Kelley, D. Knights, I. Koester, T. Kosciolek, J. Kreps, M.G.I. Langille, J. Lee, R. Ley, Y.X. Liu, E. Loftfield, C. Lozupone, M. Maher, C. Marotz, B.D. Martin, D. McDonald, L.J. McIver, A.V. Melnik, J.L. Metcalf, S.C. Morgan, J.T. Morton, A.T. Naimey, J.A. Navas-Molina, L.F. Nothias, S.B. Orchanian, T. Pearson, S.L. Peoples, D. Petras, M.L. Preuss, E. Pruesse, L.B. Rasmussen, A. Rivers, M.S. Robeson, 2nd, P. Rosenthal, N. Segata, M. Shaffer, A. Shiffer, R. Sinha, S.J. Song, J.R. Spear, A.D. Swafford, L.R. Thompson, P.J. Torres, P. Trinh, A. Tripathi, P.J. Turnbaugh, S. Ul-Hasan, J.J.J. van der Hooft, F. Vargas, Y. Vázquez-Baeza, E. Vogtmann, M. von Hippel, W. Walters, Y. Wan, M. Wang, J. Warren, K.C. Weber, C.H.D. Williamson, A.D. Willis, Z.Z. Xu, J.R. Zaneveld, Y. Zhang, Q. Zhu, R. Knight, J.G. Caporaso, Reproducible, interactive, scalable and extensible microbiome data science using QIIME 2, *Nat. Biotechnol.* **2019**, *37*(8), 852-857.

3. M. Martin, Cutadapt removes adapter sequences from high-throughput sequencing reads, *EMBnet. journal* **2011**, *17,* 10-12.

4. Callahan, B. J., Mcmurdie, P. J., Rosen, M. J., Han, A. W., Johnson, A. J., & Holmes, S. P. Dada2: high-resolution sample inference from illumina amplicon data. *Nat. Methods*, **2016**, *13*(7), 581-583.

5. Katoh, & K. Mafft: a novel method for rapid multiple sequence alignment based on fast fourier transform. *Nucleic Acids Res.* **2002**, *30*(14), 3059-3066.

6. Price, M.N., Dehal, P.S., and Arkin, A.P. FastTree: computing large minimum evolution trees with profiles instead of a distance matrix. *Mol. Biol. Evol.* **2009**, 26, 1641-1650.
